# Small PARP inhibitor PJ-34 induces cell cycle arrest and apoptosis of adult T-cell leukemia cells

**DOI:** 10.1186/s13045-015-0217-2

**Published:** 2015-10-23

**Authors:** Xue Tao Bai, Ramona Moles, Hassiba Chaib-Mezrag, Christophe Nicot

**Affiliations:** Department of Pathology and Laboratory Medicine, Center for Viral Oncology, University of Kansas Medical Center, 3901 Rainbow Boulevard, Kansas City, KS 66160 USA

## Abstract

**Background:**

HTLV-I is associated with the development of an aggressive form of lymphocytic leukemia known as adult T-cell leukemia/lymphoma (ATLL). A major obstacle for effective treatment of ATLL resides in the genetic diversity of tumor cells and their ability to acquire resistance to chemotherapy regimens. As a result, most patients relapse and current therapeutic approaches still have limited long-term survival benefits. Hence, the development of novel approaches is greatly needed.

**Methods:**

In this study, we found that a small molecule inhibitor of poly (ADP-ribose) polymerase (PARP), PJ-34, is very effective in activating S/G2M cell cycle checkpoints, resulting in permanent cell cycle arrest and reactivation of p53 transcription functions and caspase-3-dependent apoptosis of HTLV-I-transformed and patient-derived ATLL tumor cells. We also found that HTLV-I-transformed MT-2 cells are resistant to PJ-34 therapy associated with reduced cleaved caspase-3 activation and increased expression of *RelA/p65*.

**Conclusion:**

Since PJ-34 has been tested in clinical trials for the treatment of solid tumors, our results suggest that some ATLL patients may be good candidates to benefit from PJ-34 therapy.

## Introduction

Human T-cell leukemia virus type I (HTLV-I) is etiologically linked to the development of an aggressive type of peripheral T-cell leukemia known as ATLL [1]. The clinical course varies among infected patients and the disease has been classified into four distinct entities: smoldering, chronic, acute, or lymphoma [2]. Although many features of HTLV-I biology have been discovered [3], the treatment of the disease remains unsatisfactory, with minimal improvements in the overall survival of patients [4]. Overall, the current therapies used for the treatment of ATLL patients in the acute phase have limited impact and the overall projected 4-year survival rate of acute ATLL is around 5 % [5]. The mechanism by which HTLV-I causes ATLL is still not fully understood, but a latency period of several decades before the onset of the disease suggests that long-term survival and expansion of virus-infected cells are required. Along these lines, we have previously shown that reactivation of telomerase activity is one of the essential steps in the transformation process of HTLV-I-infected cells [6]. HTLV-I transformed CD4/CD25+ T cells in vivo and in vitro. In early stages, infected cells may rely on an autocrine/paracrine IL-2/IL-2R or IL-15/IL-15R cytokine loop for active proliferation [7]. During that stage, HTLV-I-infected cells accumulate genetic and epigenetic mutations and are prone to genomic instability. At the basis of this phenomenon is the viral oncoprotein Tax, which has been shown to inactivate tumor suppressors such as p16ink, p53, RB, and p21WAF [8], affect genome stability [9], and activate oncogenic signaling pathways such as NF-κB, Notch, and JAK/STAT [10–12]. In addition, Tax also induces DNA breaks during cellular replication and inhibits DNA repair pathways, leading to accumulation of genetic alterations [13, 14]. Eventually, an infected IL-2-independent transformed cell emerges with a selective growth advantage resulting in clonal expansion. The molecular basis for IL-2 independence is still unknown although a majority of HTLV-I-transformed cells simultaneously acquire constitutive JAK/STAT activation. The transition from IL-2 dependent to IL-2 independent is believed to mimic the disease progression from smoldering or chronic to the acute type of ATLL. Recently, we showed that Tax can induce genomic DNA double-strand breaks (DDSB) by targeting the fork of replication during cell division [13]. Since HTLV-I-transformed cells have a defective homologous recombination repair (HR) pathway [14], we hypothesized that HTLV-I-transformed and ATLL cells might be particularly sensitive to small drug inhibitors targeting DNA replication. Although poly (ADP-ribose) polymerase (PARP) is a single-strand break sensing protein, PARP inhibitors (PARPi) have been shown to be selectively effective in cells with an HR-defective pathway [15]. Numerous PARPi (PJ-34, MK4827, ABT-888, AZD2281, and BSI-201) are in clinical trials for breast cancer, ovarian cancer, and prostate cancer [16, 17]. The PARPi PJ-34 has been shown to cause cell cycle arrest in various human cancers, including myelodysplastic syndromes (MDS) and acute myeloid leukemia (AML) [18, 19].

In this study, we investigated the efficacy of the PARPi PJ-34 in targeting HTLV-I-transformed cells and a panel of patient-derived ATLL cell lines. Our results demonstrate that PJ-34 used as a single agent is a potent inhibitor of cellular growth in IL-2-dependent as well as IL-2-independent transformed ATLL cells. We also found that another PARPi (olaparib/AZD2281) is also effective against HTLV-I-transformed cells. We further show that cells treated with PJ-34 reactivated p53 functions and accumulated in G2/M. Tumor cells died from apoptosis as shown by annexin V staining but this process appears to be largely p53 - independent since ATLL-derived cells not expressing p53 (MT-1 and ED) were still efficiently killed by PJ-34. We found that HTLV-I-transformed MT-2 and C91PL cell lines were resistant to PJ-34 treatment. We found that PJ-34-resistant cells expressed higher basal levels of Bax and were unable to engage the cleavage of pro-caspase-3. In addition, resistance of MT-2 cells was independent from p53BP1 and PARP1 but coincides with activation of NF-κB.

## Materials and methods

### Cell lines and reagents

HTLV-I-transformed cell lines (MT-4, MT-2, C8166, C91PL) and ATL-like cell lines, IL-2 independent (MT-1, ATL-T, ED-40515(−), ALT-25), were maintained in RPMI-1640 media supplemented with 10 % FBS, penicillin, and streptomycin. ATL-like cell lines, IL-2 dependent (ATL-43T, KOB, and ATL-55T), were maintained in RPMI-1640 supplemented with 10 % FBS, penicillin, and streptomycin and IL-2 (50 U/mL). PARP-1 inhibitor, PJ-34 (N-(5, 6-Dihydro-6-oxo-2-phenanthridinyl)-2-(dimethylamino) acetamide, ab120981), was purchased from Abcam. Stock solutions (25 mM) were made with dimethyl sulfoxide (DMSO). In all experiments, cells were treated with different concentrations of PJ-34 (5, 10, 25, and 50 μM) or DMSO for 3 or 5 days, as indicated. Olaparib was used at 25 μM for 3 days. Cell Proliferation Kit II (XTT) was purchased from Roche Life Science.

### Cell cycle and cell proliferation

HTLV-I-transformed and ATL cells were treated with DMSO or PJ-34 for 3 days. The cells were collected and washed with 1× PBS and fixed with 75 % EtOH overnight at −20 °C. The next day, cells were washed with ice-cold 1× PBS, followed by treatment with RNase for 15 min at 37 °C, stained with 100 μg/mL propidium iodine (PI) for 15 min at room temperature, and analyzed by flow cytometry. Cell proliferation was measured by the XTT assay. For the XTT assay, 100 μL (10,000) cells were seeded in 96-well plates and treated with different concentrations of PJ-34 (5, 10, 25, and 50 μM) or DMSO for 5 days. A 50-μL of XTT labeling mixture was added to each well and incubated for 6 h. The absorbance was measured at 450 and 620 nm by spectrophotometry. Results were plotted as mean ± SD from at least two independent experiments.

### Cell tracking assay

CFDA-SE (Molecular Probes, Eugene, OR) was used to label cells according to the manufacturer’s protocol. After labeling, 1 × 10^5^ cells were analyzed by FACS assay for 0 h control. The rest of the labeled cells were divided into two groups: one treated with DMSO, the other one with PJ-34. Every 24 h, 1 × 10^5^ cells were taken out for FACS assay until 72 h.

### Apoptosis assays and mitochondrial membrane potential

The cells were collected and washed with 1× PBS then stained with annexin V/propidium iodide using the Alexa Fluor® 488 Annexin V/Dead Cell Apoptosis Kit (Molecular Probes, Eugene, OR) according to the manufacturer’s instructions. MT-4, C91PL, and MT-2 cells were treated with DMSO or PJ-34. Then, the cell mitochondrial membrane potential (ΔΨm) was measured using the JC-1 Assay Kit (Invitrogen) according to the manufacturer’s instructions.

### Western blotting

Total cell extracts were prepared with RIPA buffer (50 mM Tris HCl pH 8.0, 150 mM NaCl, 1 % NP-40, 0.5 % sodium deoxycholate, 0.1 % SDS). Samples were separated on SDS-PAGE followed by electroblotting to polyvinylidene difluoride membranes. The following antibodies were used: cyclin E (C-19), cyclin A (h432), cyclin B1 (H-20), p21 (C-19), actin (C-11) and p53 (FL-393), Bcl-2 (100), Bax (N-20), Bcl-xS/L (S-18), caspase-8 (H-134), and caspase-3 (H-60) are all from Santa Cruz Biotechnology; Phospho-Histone H2A.X (Ser139) (p-H2AX, 2577) and Phospho-p53 (Ser15) (pS15-p53, D4S1H) are from Cell Signaling Biotechnology.

### Real-time RT-PCR

Total RNA was prepared with TRIzol reagent (Invitrogen, Carlsbad, CA). After DNase I treatment, the RNA was reverse transcribed and the cDNA was used for PCR and real-time PCR. The real-time PCR assay was performed using the iTaq™ Universal SYBR® Green Supermix. Real-time PCR was used to detect the expression of Bax, MDM2, GADD45α, XIAP, FLIP, p53BP1, BRCA1, p65/RELA, and PARP1. Real-time PCR was performed in duplicate, and data were normalized to GAPDH expression.

## Results

### PJ-34 induces cell cycle arrest in HTLV-I-transformed and ATLL-derived cell lines

**Fig. 1:**
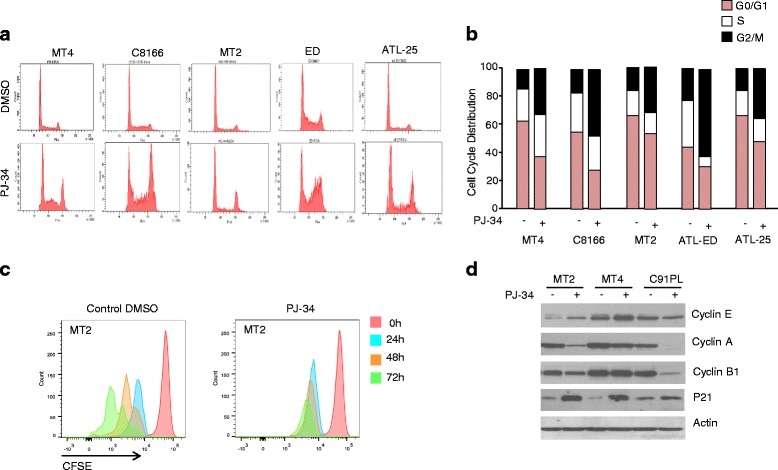
PJ-34 induces S/G2M cell cycle arrest. **a** Cell cycle analysis of HTLV-I-transformed and ATL cells after DMSO or PJ-34 treatment. **b** Cell cycle profile of DMSO- or PJ-34-treated cells. **c** Cell tracking of MT-2 with CFDA-SE after DMSO or PJ-34 treatment. Representative results are shown here. **d** Western blot of cyclin E, cyclin A, cyclin B1, and p21 in MT-2, MT-4, and C91PL cells after DMSO or 25 μM PJ-34 treatment. Actin was used to confirm equal loading

HTLV-I-transformed and ATLL cells are characterized by excessive chromosomal instability associated with defects in HR DNA repair pathways. We, therefore, hypothesized that these cells might be particularly sensitive to PARPi. HTLV-I-transformed T-cell lines MT-2, MT-4, C8166, and patient-derived ATLL cell lines ED and ATL-25 were treated with PJ-34 for 3 days and cell cycle was analyzed by propidium iodide staining and DNA content analyses on live gated cells by FACS. Our results revealed a marked increase of cells in the G2/M phase of cell cycle following treatment (Fig. 1a, b). These results are consistent with previous findings that the mitotic spindle checkpoint is functional in HTLV-I-transformed and Tax-expressing cells, which arrest in G2/M following treatment with NU7026, Taxol, or Nocodazole [20, 21]. We next used carboxyfluorescein succinimidyl ester (CFSE) incorporation and confirmed that HTLV-I-transformed MT-2 cells treated with PJ-34 are no longer dividing (Fig. 1c), suggesting that these cells are arrested in all the different phases of the cell cycle. Consistent with these results, we observed a significant increase in cyclin-dependent kinase inhibitor (CDK) p21WAF expression in MT-2, C91PL, and MT-4 cells after treatment with PJ-34 (Fig. 1d). Cyclin A associates with CDK2 and is involved in the initiation and completion of DNA replication during S phase [22]. Our experiments revealed a significant decrease in cyclin A expression after treatment with PJ-34. Since cyclin A is also important for centrosome replication and G2/M transition [23], these data may provide an explanation for G2/M arrest observed in most cells after treatment with PJ-34. In contrast, cyclin E, involved in G1 exit and initiation of S phase [24], was downregulated by PJ-34 in C91PL cells only (Fig. 1d). We next investigated the expression of Cyclin B1 because of its role in mitosis [25]. We found that cyclin B1 expression was significantly reduced in MT-2 and C91PL but not in MT-4 transformed cells (Fig.1d). Overall, our results suggest that PARPi PJ-34 affects multiple cyclins and is effective in preventing proliferation of HTLV-I-transformed cells.

**Fig. 2:**
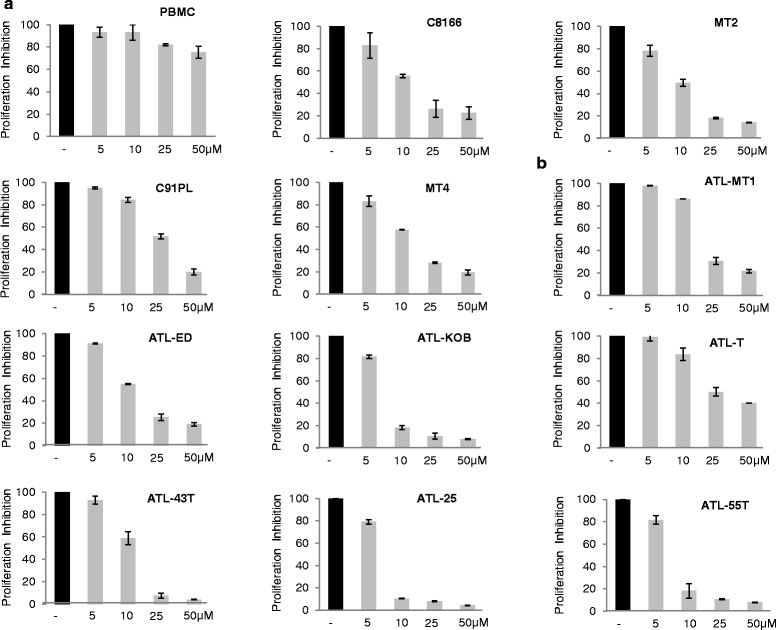
**a** PBMCs, HTLV-1-transformed (C8166, MT-2, C91PL, MT4), and (**b**) ATL cells (ATL-MT-1, ATL-ED, ATL-KOB, ATL-T, ATL-43T, ATL-25, ATL-55T) were treated with DMSO or different concentrations of PJ-34 (5, 10, 25, 50 μM) for 5 days; cell proliferation was measured using XTT assay. Results were plotted as mean ± SD from at least two independent experiments

Next, we measured quantitative inhibition of cellular proliferation using XTT assays, a colorimetric assay for the quantification of cellular proliferation. These assays were performed in four HTLV-I-transformed cells and seven ATLL-derived cell lines and a dose increase of PJ-34 (5–50 μM) or DMSO vehicle as a control (Fig. 2). To demonstrate specificity of PJ-34, we used human PBMCs from a HTLV-I-negative healthy donor. These studies demonstrate that IC50 for most HTLV-I-transformed or ATL cells is around 10 μM (MT-1 and C91PL 20 μM) (Fig. 2). Even at 50 μM of PJ-34, PBMCs present less than 25 % inhibition when most HTLV-I-transformed cells had more than 80 % inhibition (Fig. 2).

### PJ-34 induces p53-dependent and p53-independent apoptosis in HTLV-I-transformed and ATLL-derived cell lines

**Fig. 3:**
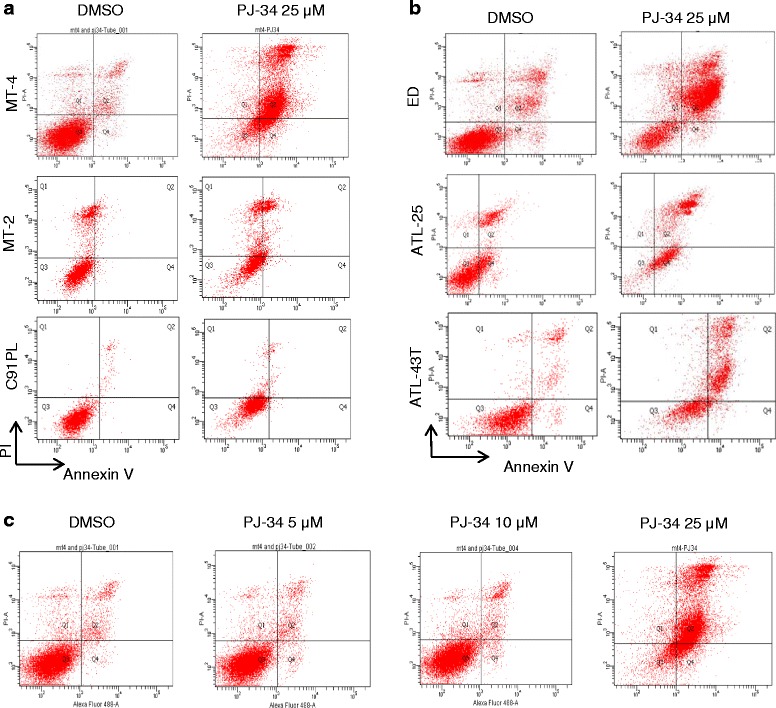
PJ-34 induces cell apoptosis in most HTLV-I-transformed and ATL cells except for C91PL and MT-2 cells. **a** HTLV-1-transformed and (**b**) ATL cells were treated with 25 μM PJ-34 for 3 days. Cells were subsequently stained for annexin V and PI and analyzed for apoptosis by FACS analysis. **c** MT-4 cell line was treated with DMSO or different amounts of PJ-34 (5, 10, 25 μM). Cells were subsequently stained for annexin V and PI and analyzed for apoptosis by FACS analysis

**Fig. 4:**
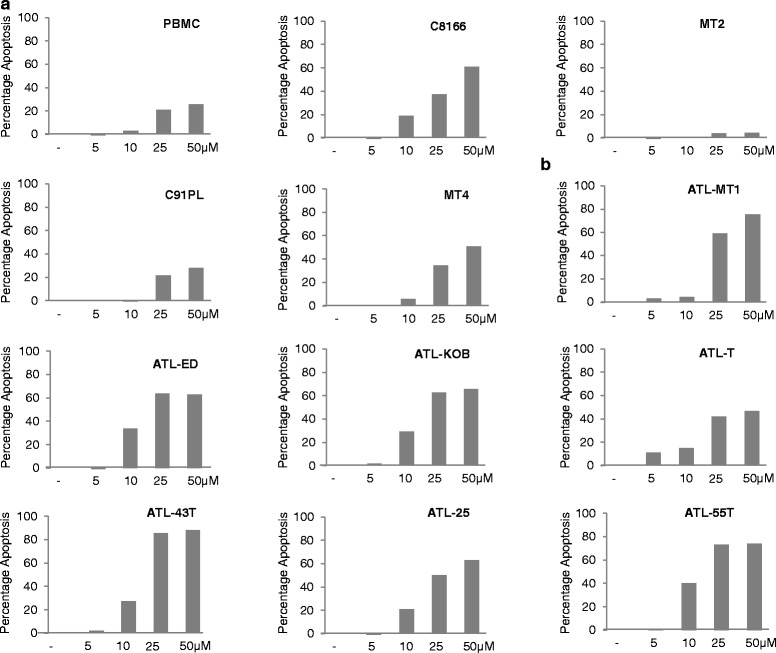
PJ-34 causes DNA damage and p53 pathway activation, even in MT-2 and C91PL cells. PJ-34 induces cell apoptosis in most of HTLV-1-transformed and ATL cells except for C91PL and MT-2 cells. **a** PBMCs, HTLV-1-transformed (C8166, MT-2, C91PL, MT4), and (**b**) ATL cells (ATL-MT-1, ATL-ED, ATL-KOB, ATL-T, ATL-43T, ATL-25, ATL-55T) were treated with DMSO or different concentrations of PJ-34 (5, 10, 25, 50 μM) for 5 days. Cells were subsequently stained for annexin V and PI and analyzed for apoptosis by FACS analysis. Percentage of increased apoptotic and necrotic cells in HTLV-1-transformed cells and ATL cell treatment with different amounts of PJ-34 were graphed

**Fig. 5:**
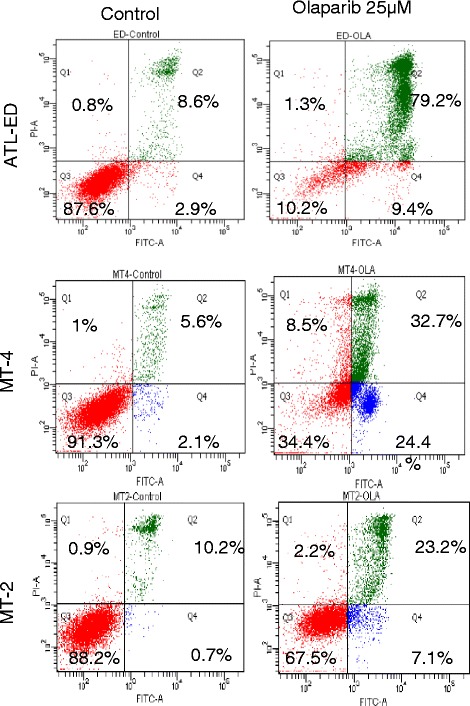
PARPi olaparib (AZD2281) was used at 25 μM for 3 days on HTLV-I-transformed MT-4, C91PL and MT-2 cells and apoptosis was measured by annexin V staining and FACS

PARPi have been used to induce apoptosis in breast cancer and ovarian cancer cells [26–28]. The lack of effective therapy for ATLL prompted us to investigate the cytotoxicity of PJ-34 in four HTLV-I-transformed and seven ATLL-derived cell lines. Cells were treated for 5 days and analyzed by annexin V staining. Our results demonstrated a significant level of apoptosis in all patient-derived ATLL cell lines and most HTLV-I-transformed cell lines, with the exception of C91PL and MT-2 which appear to be resistant to PJ-34-induced apoptosis (Fig. 3a, b). These results were confirmed using a dose-dependent increase of PJ-34 on HTLV-I-transformed MT-4 cells (Fig. 3c). To confirm specific effects of PJ-34 on HTLV-I-transformed cells, we used a dose-dependent increase of PJ-34 and normal human PBMCs as a negative control (Fig. 4). Overall, these studies confirmed our previous observations and showed that HTLV-I-transformed cells are more sensitive than PBMC controls (Fig. 4). Also in agreement with above-mentioned data, C91PL had kinetics similar to PBMC and MT-2 was resistant to PJ-34 even at a dose of 50 μM (Fig. 4). Interestingly, another PARPi, olaparib (AZD2281), also induced apoptosis in HTLV-I-transformed MT-4 and ATL-ED resulting in only 34 and 10 % live cells, respectively, after 3 days of treatment. In contrast, similar to PJ-34, MT-2 cells were more resistant, with 67 % live cells after 3 days of olaparib (Fig. 5).

### PJ-34 treatment leads to reactivation of p53 functions in HTLV-I-transformed cells

**Fig. 6:**
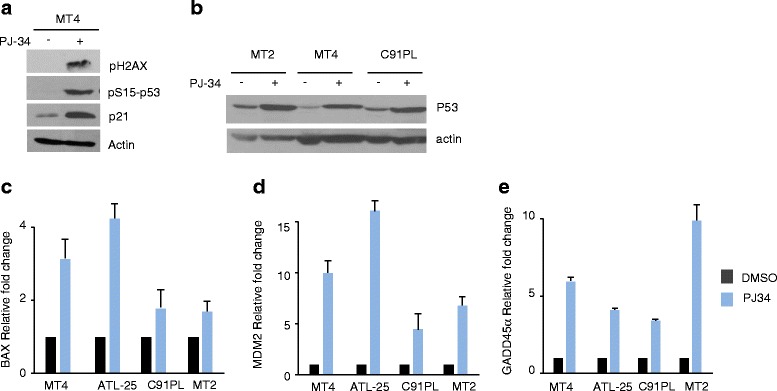
PJ-34 causes DNA damage and p53 pathway activation even in MT-2 and C91PL cells. **a** Western blot of p-H2AX, pS15-p53 and p21 in MT-4 cells treated with DMSO or PJ-34. Actin was used to confirm equal loading. **b** Western blot of p53 in MT-2, MT-4 and C91PL cells after treatment with DMSO or PJ-34. **c**–**e** Cells were treated with DMSO or PJ-34 and the changes of p53 target genes Bax (**c**), MDM2 (**d**), and GADD45a (**e**) were analyzed using real-time RT-PCR. Real-time PCR was performed in duplicate, and data were normalized to GAPDH expression. Results were presented as mean ± SD from two independent experiments

As a result of PARP inhibition by PJ-34, unrepaired single-strand DNA breaks (SSB) are converted into double-strand breaks (DDSB) at fork replication and result in accumulation of DDSB and p-H2AX foci. Consistent with this notion, we found an increased p-H2AX expression in cells treated with PJ-34 (Fig. 6a). Accumulation of p-H2AX is known to result in ATM/ATR activation and downstream Chk1-p53 pathway. Indeed, treatment of HTLV-I-transformed cells with PJ-34 caused an increased p-p53 at serine 15 and p21WAF (Fig. 6a), suggesting that p53 may be active in these cells. Drug cytotoxicity is often associated with activation of p53-dependent apoptosis, and tumors with mutated inactive p53 are frequently resistant to radio- and chemotherapy. HTLV-I-transformed cells in vitro and in vivo have been shown to express high levels of p53 that is genetically or functionally inactivated [29–31] but can be reactivated leading to either apoptosis or senescence of HTLV-I-transformed cells [32]. We compared activation of p53-mediated transcription in MT-4 (PJ-34 sensitive) and C91PL or MT-2 (PJ-34 resistant). Our results suggested that p53 was functionally reactivated in all HTLV-I-transformed cells following treatment with PJ-34, as demonstrated by an increase in p53 (Fig. 6b) and increased mRNA expression of the p53 target genes Bax, MDM2, p21, and GADD45α (Fig. 6c–e). One common target of both p53-dependent G1 arrest and p53-independent G2 arrest is p21WAF, whose expression was consistently increased after PJ-34 treatment of HTLV-I-transformed cells. However, our studies suggest that the sensitivity of HTLV-I-transformed cells to PJ-34-induced apoptosis may not be dependent upon p53 signaling since both MT-2 and C91PL cells were resistant to PJ-34-mediated apoptosis despite reactivation of p53 in these cells. In addition, some ATLL-derived HTLV-I-transformed cells such as ED or MT-1 have no detectable expression of functional p53 and yet these cells were sensitive to PJ-34 treatment (Figs. 2 and 4).

### Resistance of HTLV-I-transformed cells to PJ-34-induced apoptosis is associated with defective caspase-3 activation

**Fig. 7:**
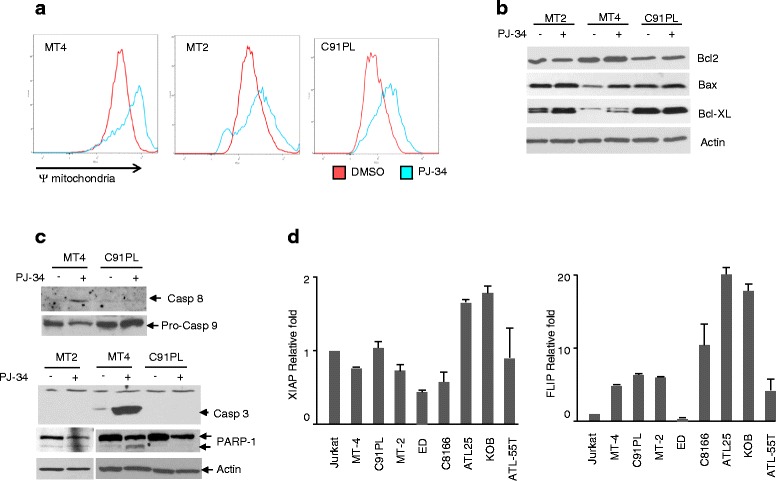
The defect of caspase-3 activation in MT-2 and C91PL contributes to the resistance of PJ-34 in MT-2 and C91PL cells. **a** MT-4 and MT-2 were treated with DMSO or PJ-34. Cells were subsequently stained with JC-1, and mitochondrial membrane potential (ΔΨm) was measured by FACS analysis. Representative results of two experiments are shown here. **b** Western blot of analysis of Bcl-2, Bax, and Bcl-xL in MT-2, C91PL, and MT-4 after treatment with DMSO or PJ-34. **c** MT-2, MT-4, and C91PL were treated with DMSO or PJ-34 for 3 days. Then, total cell extracts were probed for caspase-8, caspase-3, and PARP-1 expression. **d** Relative expression of FLIP and XIAP were compared using real-time RT-PCR among resistant cell lines MT-2, C91PL, and other sensitive cell lines. Results were presented as mean ± SD from two independent experiments

Disruption of the mitochondrial membrane potential, ΔΨm, represents a critical step in the activation process of apoptosis cell death. Therefore, we measured ΔΨm in MT-4, MT-2, and C91PL cells treated with PJ-34. Our results suggested that the ΔΨm collapse was pronounced when HTLV-I-transformed cells were treated with PJ-34, and MT-4 cells were significantly more affected than MT-2 (Fig. 7a). The collapse of the ΔΨm is usually associated with activation of caspases. Caspase-9 is activated immediately downstream of the mitochondria following cytochrome C release and apoptosome formation. In contrast, caspase-8 is traditionally activated by death receptor signaling. These are referred to as canonical and non-canonical apoptosis pathways [33]. Caspase-3 is a central mediator in the caspase pathway and poly (ADP-ribose) polymerase (PARP) is a substrate of caspase-3 protease activity and its processing is usually associated with apoptotic cell death. To gain further insight into the mechanism used by MT-2 and C91PL-transformed cells to resist PJ-34 toxicity, we analyzed different markers associated with pro-apoptotic and anti-apoptotic activities. Although Bcl-xL is overexpressed and an important anti-apoptotic factor in HTLV-I cells [34], the surge in levels of Bax expression cannot be counteracted by high levels of Bcl-xL, since the latter does not prevent Bax-mediated apoptosis [35]. Expression of anti-apoptotic Bcl-2 was largely unchanged after treatment with PJ-34 (Fig. 7b). We then evaluated whether the observed apoptotic phenotype of HTLV-I-transformed cells treated with PJ-34 was dependent upon the mitochondrial pathway by assessing Bax activation and activation of caspase-3. Surprisingly, active caspase 3 cleaved products were readily detected in MT-4-treated cells but not detected in MT-2 and C91PL cells after exposure to PJ-34 (Fig. 7c). Therefore, we investigated levels of FLIP and XIAP since these are known to affect activation of caspase-3, but no difference was detected for MT-2 and C91PL cells (Fig. 7d). We amplified and sequenced the cDNA of the caspase-3 gene, but it was wild type in both MT-2 and C91PL. The reason for the lack of active caspase-3 in MT-2 and C91PL after PJ-34 treatment is still unclear at the moment and under further investigation.

### Resistance to PJ-34 in HTLV-I-transformed MT-2 cells coincides with increased expression of RelA/p65

**Fig. 8:**
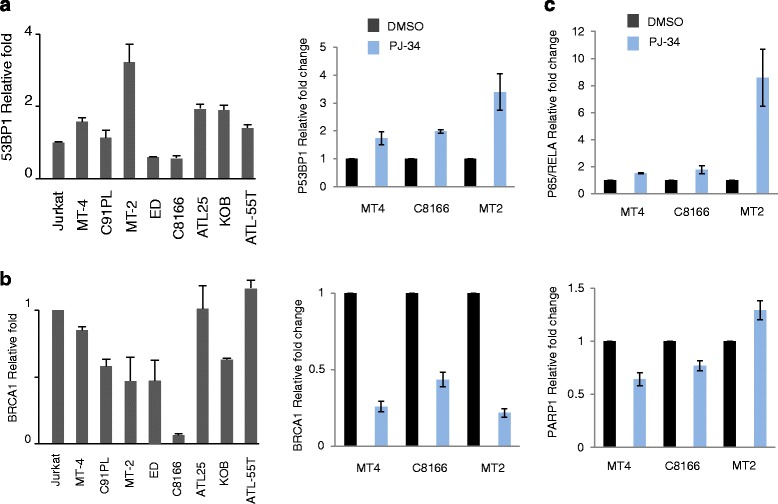
The expressions of known resistant markers in MT-2 and sensitive cell lines. (The defect of caspase 3 activation in MT-2 and C91PL contributes to the resistance of PJ-34 in MT-2 and C91PL cells.) **a**, **b** Relative expression of p53BP1 (**a**) and BRCA1 (**b**) was compared using real-time RT-PCR among resistant cell lines MT-2, C91PL, and other sensitive cell lines. Real-time PCR was performed in duplicate, and data were normalized to GAPDH expression. Results were presented as mean ± SD from two independent experiments. **c** Relative expression of p53BP1, p65/RELA, BRCA1, and PARP-1 was compared using real-time RT-PCR among MT-4, C8166, and MT-2 treated with DMSO or 25 μM of PJ-34 for 5 days. Real-time PCR was performed in duplicate, and data were normalized to GAPDH expression

We next wanted to understand the molecular mechanism involved in the resistance of HTLV-I-transformed cells MT-2 and C91PL. This has direct and important implications for treatment of ATLL patients with PJ-34, and identification of resistance biomarkers could also provide new insights for PARPi treatment of breast, ovarian, and prostate cancer patients. Acquisition of resistance to PARPi can be achieved by genetic alterations that restore HR functions [36]. Frequently, increased expression of BRCA1 or p53BP1 has been shown to correlate with resistance to PARPi [37, 38]. However, expression levels of p53BP1 or BRCA1 were not affected in HTLV-I-transformed MT-2 or C91PL before or after PJ-34 treatment (Fig. 8a, b). Increased expression of miR-107 was associated with PARPi sensitivity [39]. However, analyses of mature miR-107 expression revealed a significant increased expression in both resistant and sensitive HTLV-I-transformed cells (except for ED) when compared with HTLV-I-negative Jurkat T cells (data not shown). Similarly, the expression of PARP1 was not affected in MT-2-treated cells (Fig. 8c). In contrast, a significant increase in RelA/p65 was detected specifically in MT-2 but not MT-4 or C8166 cells following treatment with PJ-34, suggesting that canonical NF-κB activation may play an important role in resistance to PARPi in these cells.

## Discussion

ATLL has a poor clinical outcome and current therapies have limited long-term benefits, in addition to most patients with acute or lymphoma ATLL having a short relapse-free survival. Patients diagnosed with chronic or smoldering forms of ATLL have a longer life expectancy but current treatments are ineffective and patients eventually progress to the acute type. Numerous studies have established that cancers with a dysfunction of the HR DNA repair pathway, also referred to as “BRCAness,” share characteristics with BRCA1- or BRCA2-mutated cancer cells and are very sensitive to PARPi [40, 41]. We have previously reported that HTLV-I-transformed cells have pronounced defects in the homologous recombination (HR) DNA repair pathway. Additional studies demonstrated that tumor cells with a defective base excision repair (BER) pathway are prone to DDSB accumulation and hypersensitive to PARP targeting [42]. Notably, HTLV-I-transformed cells expressing the viral oncoprotein Tax have a defective BER pathway [43, 44]. These observations prompted us to investigate the potential benefits of PARPi therapy for ATLL.

In this study, we found a potent anti-proliferative effect of PARP small inhibitor PJ-34 against HTLV-I-transformed cells and patient-derived ATLL cells. Reduced tumor cell proliferation was demonstrated using XTT and CFSE staining assays. We showed that HTLV-I-transformed cells arrested in the G2/M phase of the cell cycle following treatment with PJ-34 was consistent with alterations in cyclins and cyclin-dependent kinase inhibitor (CDKI) expression. The effect of PJ-34 was time- and dose- dependent and affected both IL-2-dependent and IL-2-independent HTLV-I-transformed cells. We further demonstrated that PJ-34 is very effective against a panel of patient-derived ATLL cell lines, suggesting therapeutic potential. In general, anti-proliferative effects were associated with apoptotic cell death as shown by annexin V staining and the activation of caspase-3. We detected a loss of the mitochondrial membrane potential in HTLV-I cells following PJ-34 treatment, supporting the notion that PJ-34 induces cell death via the intrinsic mitochondrial pathway. Interestingly, treatment with PARPi PJ-34 resulted in accumulation of DNA breaks and reactivation of the tumor suppressor p53 transcriptional functions, as demonstrated by an increased expression of target genes p21WAF, MDM2, BAX, and GADD45. However, reactivation of p53 was not critical for PJ-34’s effects since ATLL ED cells do not express p53 and were still sensitive to PJ-34. Interestingly, our studies identified two HTLV-I-transformed cell lines that were resistant to PJ-34 treatment: MT-2 and C91PL. We then investigated differences between MT-4 (PJ-34 sensitive) and MT-2 and C91PL (PJ-34 resistant). We found no specific pattern of expression for p53BP1 or BRCA1 in PJ-34-resistant versus sensitive cells. We then investigated expression of pro- and anti-apoptotic factors. Overall, anti-apoptotic proteins were not differentially affected but only MT-4 cells showed an increase in pro-apoptotic Bax expression. Bcl-xL, a pro-survival member of the Bcl-2 family, cannot directly inhibit Bax and specifically inhibits certain upstream BH3-only proteins. This supports our observation that the upregulation of Bcl-xL seen in MT-4 cells is unable to inhibit PJ-34-induced apoptosis and Bax activation, leading to apoptosis. We found that PJ-34-resistant cells were unable to activate caspase-3. This was unexpected because we and others previously demonstrated that MT-2 cells can activate caspase-3-dependent apoptosis [45–47].

Recently, PARP1 and PARP2 were found to play a role in sensing stalled or collapsed replication forks and to recruit the Mre11-Rad50-NBS1 (MRN) complex for resection and single-stranded DNA (ssDNA) formation, which allows RAD51 binding onto resected DNA to initiate HR [48, 49]. Thus, PARP is also involved in HR repair at replication forks and inhibition of PARP leads to increased DNA lesions that can cause stalling and collapse of the DNA replication machinery. These observations suggest that targeting PARPi may be a good approach for ATLL patients. Combination therapy using PJ-34 and DNA-damaging agents such as cisplatin has shown promising results in other cancers and may be considered for a phase I trial of HTLV-I-associated ATLL. The reproducible growth arrest in cells from different genetic backgrounds highlights the potential value of PARPi as therapeutic agents for the treatment of ATLL.
